# Multi-Target HCC Blood Test Demonstrates Consistent Performance Across Subgroups of Patients with Chronic Liver Disease

**DOI:** 10.3390/cancers18172777

**Published:** 2026-08-27

**Authors:** Amit G. Singal, Mark Camardo, Janelle J. Bruinsma, Elle Kielar-Grevstad, Naga Chalasani, Binu V. John

**Affiliations:** 1 Department of Internal Medicine, UT Southwestern Medical Center, Dallas, TX 75390, USA; 2 Abbott, Madison, WI 53719, USA; 3 Department of Internal Medicine, Indiana University, Indianapolis, IN 46202, USA; 4 Department of Medicine, University of Miami Miller School of Medicine, Miami, FL 33136, USA; 5 Division of Gastroenterology and Hepatology, Miami VA Health System, Miami, FL 33136, USA

**Keywords:** ultrasound, mt-HBT, screening, surveillance, liver cancer

## Abstract

Background: Early detection of hepatocellular carcinoma (HCC) is critical for improving patient outcomes; however, ultrasound-based surveillance has limited sensitivity and variable performance across patient populations. We evaluated the performance of a multitarget HCC blood test (mt-HBT), incorporating methylated DNA markers, alpha-fetoprotein (AFP), and patient sex, across clinically relevant subgroups. Methods: We performed a subgroup analysis of a multicenter, prospective case-control study that included 159 patients with early-stage HCC (Barcelona Clinic Liver Cancer Stage 0/A) and 649 control patients with cirrhosis or chronic hepatitis B without HCC. The mt-HBT combined methylated HOXA1, TSPYL5, and B3GALT6 markers with AFP and sex. Sensitivity and specificity were evaluated overall and according to age, sex, obesity, liver disease etiology, Child Pugh class, and tumor size. Performance was compared with AFP and GALAD. Results: Overall sensitivity and specificity of mt-HBT for early-stage HCC detection were 76.7% (95% CI, 69.6–82.6) and 87.5% (95% CI, 84.8–89.8), respectively. Sensitivity was significantly higher than that of AFP (35.2%, *p* < 0.001) and comparable to that of GALAD (78.6%, *p* = 0.56), whereas specificity was lower than that of AFP (98.5%, *p* < 0.001) but higher than that of GALAD (76.9%, *p* < 0.001). Sensitivity was maintained across key subgroups, including patients with obesity (68.9%), Child Pugh B cirrhosis (75.0%), hepatitis C (80.0%), hepatitis B (72.2%), alcohol-associated liver disease (80.5%), and metabolic dysfunction-associated steatotic disease (69.0%) (all *p* > 0.05 between subgroups). Specificity exceeded 80% in all examined populations and was significantly higher in women than in men (92.5% vs. 83.9%, *p* < 0.001). Sensitivity increased with tumor size, ranging from 60.0% for tumors < 2 cm to 100% for tumors > 5 cm. Conclusions: The mt-HBT demonstrated robust, consistent performance for early-stage HCC detection across diverse patient populations, including subgroups in which ultrasound surveillance commonly underperforms. These findings support the prospective validation of mt-HBT as a blood-based surveillance strategy for HCC.

## Simple Summary

The suboptimal sensitivity of ultrasound-based surveillance underscores a need for new surveillance strategies. Herein, we report subgroup analyses of a multicenter, prospective case-control study that included 159 patients with early-stage HCC and 649 control patients with cirrhosis or chronic hepatitis B without HCC. Overall sensitivity and specificity of mt-HBT for early-stage HCC detection were 76.7% (95% CI, 69.6–82.6) and 87.5% (95% CI, 84.8–89.8), respectively. Sensitivity was maintained across key subgroups, including patients with obesity, Child–Pugh B cirrhosis, hepatitis C, hepatitis B, alcohol-associated liver disease, and metabolic dysfunction-associated steatotic disease. Specificity exceeded 80% in all examined populations. Sensitivity increased with tumor size, ranging from 60.0% for tumors < 2 cm to 100% for tumors > 5 cm. In conclusion, the mt-HBT demonstrated robust, consistent performance for early-stage HCC detection across diverse patient populations, including subgroups in which ultrasound surveillance commonly underperforms.

## 1. Introduction

Hepatocellular carcinoma is the third leading cause of cancer-related death worldwide and a leading cause of death in patients with cirrhosis [1,2,3]. Further, the number of new liver cancer cases is expected to double from 0.87 million in 2022 to 1.52 million in 2050 if there is no change in current trends. Although HCC continues to have an incidence-to-mortality ratio that approaches 1, this is partly due to frequent late-stage diagnosis. Indeed, the prognosis of patients with HCC is largely dependent on tumor stage at diagnosis, with curative options, including liver transplantation and surgical resection, affording long-term survival for patients diagnosed at an early stage [4,5,6]. Conversely, patients diagnosed at later stages are typically treated with palliative locoregional or systemic therapies, and median survival is only two to three years [7,8,9,10]. Therefore, professional society guidelines recommend semi-annual HCC surveillance in at-risk individuals, including patients with chronic hepatitis B virus infection and those with cirrhosis from any etiology [11,12,13].

Ultrasound plus alpha-fetoprotein (AFP) has been the cornerstone of hepatocellular carcinoma (HCC) surveillance for over two decades. However, this strategy is limited by frequent underuse in practice and suboptimal sensitivity for early-stage HCC detection [14,15]. Ultrasound-based surveillance is performed in fewer than 30% of at-risk individuals due to a combination of patient- and provider-level barriers [14,16]. Patients report barriers including difficulty with scheduling, transportation, and costs of testing. Providers report barriers including limited time in clinic and competing clinical concerns. Further, ultrasound-based surveillance has a sensitivity of 63% for early-stage HCC detection, missing over one-third of HCC cases at an early stage [15]. Ultrasound is also recognized as being operator-dependent, with worse performance being observed in low-volume centers. Ultrasound visualization is impaired in patients with obesity and non-viral etiologies of liver disease, suggesting that ultrasound performance will further decline in contemporary populations [17].

Accordingly, interest in blood-based biomarkers has increased given their potential to address both issues [18]. Indeed, blood-based biomarkers can be performed on the same day as a clinic visit, thereby decreasing patient-level barriers and likely increasing adherence. Initial skepticism regarding blood-based biomarkers due to AFP’s low sensitivity for early-stage HCC has been mitigated by leveraging multi-biomarker panels that can improve sensitivity for early-stage detection [19]. A recent meta-analysis suggested that biomarker combinations including AFP and DCP, with or without AFP-L3, may have similar, if not higher, sensitivity than ultrasound [20].

Further, liquid biopsy biomarkers have also shown great promise for improving HCC surveillance efficacy [21]. There has been increasing interest in liquid biopsy strategies, including methylated DNA panels, extracellular vesicles, and fragmentomics [21,22,23,24,25,26]. Liquid biopsy strategies have been proposed across the cancer care continuum, including risk stratification, early detection, diagnosis, prognostication, and minimal residual disease.

Indeed, a multi-targeted HCC blood test (mt-HBT), which combines three methylation markers (*HOXA1*, *TSPYL5*, and reference marker *B3GALT6*), one protein biomarker (AFP), and patient sex, was demonstrated to have high sensitivity and specificity for early-stage HCC in a multicenter case-control study [26,27]. Subsequently, this panel was shown to have higher sensitivity for early-stage HCC than ultrasound in ALTUS, a prospective cross-sectional study among 2467 patients, of whom 40 developed HCC [28]. In the ALTUS study, the sensitivity of mt-HBT for detecting early-stage HCC was 66.7% (95% CI, 47.8 to 81.4), compared to 22.2% (95% CI, 10.6 to 40.8) for ultrasound. The specificities of mt-HBT and ultrasound were 81.9% (95% CI, 80.3 to 83.4) and 98.6% (95% CI, 98.0 to 99.0), respectively. These data underscore the promising performance of mt-HBT. However, given the relatively limited number of cases in the ALTUS study, subgroup analyses provided limited insight into potential differences in performance across patient demographics and clinical phenotypes. Herein, we report the performance of the mt-HBT panel from the previously reported case-control study across several patient subgroups of interest.

## 2. Methods

### 2.1. Patient Population

As previously described [26], mt-HBT was evaluated in a multicenter prospective case-control study. Patients were enrolled across 47 clinical sites in the United States, Europe, and Asia. Cases were defined as adult (≥18 years) patients with treatment-naïve HCC, diagnosed per LIRADS criteria on multiphase contrast-enhanced CT or contrast-enhanced MRI, or by histological confirmation. On imaging, HCC was diagnosed as a lesion ≥ 1 cm with arterial-phase hyperenhancement plus delayed washout or capsule appearance. Controls were patients with cirrhosis or chronic hepatitis B undergoing surveillance, without evidence of HCC on liver imaging (ultrasound, CT, or MRI) within 3 months of enrollment. Cirrhosis status and underlying liver disease etiology were determined according to the enrolling site’s standard clinical assessment and recorded at study enrollment [26]. All study sites had IRB approval, and informed consent was obtained from all individuals. The study was conducted in accordance with the ethical principles of the Declaration of Helsinki. The study protocol (Study 2017-01; IRB Tracking No. ESCI-18-037) was reviewed and approved by the Copernicus Group Institutional Review Board (CGIRB).

As previously described [26], algorithm development was performed in 136 HCC cases (81 early-stage) and 404 controls, matched for age and liver disease etiology. Using logistic regression analysis, three methylated markers (HOXA1, TSPYL5, and B3GALT6) plus AFP and patient sex were selected. Clinical validation was conducted in the remaining 156 HCC cases (78 early-stage) and 245 control subjects. There were no overlapping patients between the algorithm development and clinical validation groups; both groups were included in this reported analysis.

### 2.2. Mt-HBT Testing

Venous whole blood specimens were collected in LBgard^®^ Blood Tubes and BD Vacutainer^®^ Serum Separation Tubes for plasma and serum analyses, respectively. Blood specimens were processed, blinded to patient clinical information and diagnostic results. In brief, cell-free DNA was extracted from 5–6 mL of plasma using the QIAsymphony SP and QIAsymphony DSP circulating DNA kit (Qiagen, Hilden, Germany) followed by bisulfite conversion on the Hamilton Microlab STARlet (Hamilton Robotics, Reno, NV, USA) using reagents manufactured by Exact Sciences (Madison, WI, USA). Two methylation DNA markers (HOXA1 and TSPYL5) and one constitutively methylated reference marker (B3GALT6) were quantified using the Long-probe Quantification Amplified Signal (LQAS) chemistry in one triplex assay. The LQAS assay was run on a Quantstudio 5 Real-Time PCR Instrument (Life Technologies Corporation, Grand Island, NY, USA) with reagents from Exact Sciences Corporation. AFP was measured from serum via electrochemiluminescence immunoassay on the cobas^®^ e 411 instrument (Roche Diagnostics, Indianapolis, IN, USA).

### 2.3. Statistical Analysis

For this analysis, we included all control patients but focused on the subset of cases with early-stage HCC, defined as Barcelona Clinic Liver Cancer (BCLC) stage 0 or A [11]. For our primary outcome, we reported sensitivity of mt-HBT for early-stage HCC detection, overall and across subgroups by age (<65 vs. ≥65 years), sex (male vs. female), obesity (BMI < 30 vs. ≥30), liver disease etiology (hepatitis C, hepatitis B (HBV) vs. alcohol-associated liver disease (ALD) vs. metabolic dysfunction-associated steatotic liver disease (MASLD)), and liver disease severity (Child Pugh A vs. Child Pugh B). We also performed subgroup analyses evaluating the sensitivity of mt-HBT across various tumor sizes. As secondary outcomes, we also reported specificity overall and across subgroups. All estimates were paired with 95% Wilson score confidence intervals. Chi-square tests were used to compare the sensitivity and specificity of the mt-HBT across subgroups. Given the paired nature of the data, we used McNemar’s chi-square tests to compare mt-HBT’s sensitivity and specificity with AFP and GALAD, using published thresholds of 20 ng/mL and −1.36, respectively [29]. Although studies have suggested that a lower threshold for AFP may improve sensitivity for early-stage HCC, this alternative cut-off has yet to be adopted in practice or guidelines [11,12,13,30,31]. Statistical significance was defined as *p* < 0.05. No adjustments were made for multiplicity, and all results are exploratory in nature. Statistical analysis was performed using SAS EG (8.6).

## 3. Results

### 3.1. Patient Characteristics

Characteristics of the 159 cases with early-stage HCC and 649 controls with chronic liver disease are described in Table 1. The median age of patients was 63 years, and 61.5% were men. As expected, cases were slightly older (median 64 vs. 62) and had a higher male predominance (74.8% vs. 58.2%) than controls. The most common liver disease etiologies were hepatitis C virus (36.9%), MASLD (22.5%), and ALD (20.9%). Compared with controls, cases had a higher proportion of hepatitis C infection (40.9% vs. 35.9%) and ALD (25.8% vs. 19.7%), with a lower proportion of MASLD (18.2% vs. 23.6%). Median BMI was 28.5, with 41.8% being classified as obese (BMI ≥ 30). Most patients (93.2%) had cirrhosis, with 74.1% classified as Child Pugh A.

### 3.2. Overall Performance for Early-Stage HCC

The sensitivity and specificity of mt-HBT for early-stage HCC, overall and across subgroups, are reported in Table 2. The overall sensitivity and specificity of mt-HBT for early-stage HCC were 76.7% (95% CI, 69.6–82.6) and 87.5% (95% CI, 84.8–89.8), respectively. The sensitivity of mt-HBT was significantly higher than that of AFP (35.2%, 95% CI, 28.2–42.9; *p* < 0.001) but did not significantly differ from that of GALAD (78.6%, 95% CI, 71.6–84.3; *p* = 0.56). The specificity of mt-HBT was lower than that of AFP (98.5%, 95% CI, 97.2–99.2; *p* < 0.001) but higher than that of GALAD (76.9%, 95% CI, 73.5–80.0; *p* < 0.001).

**Figure 1:**
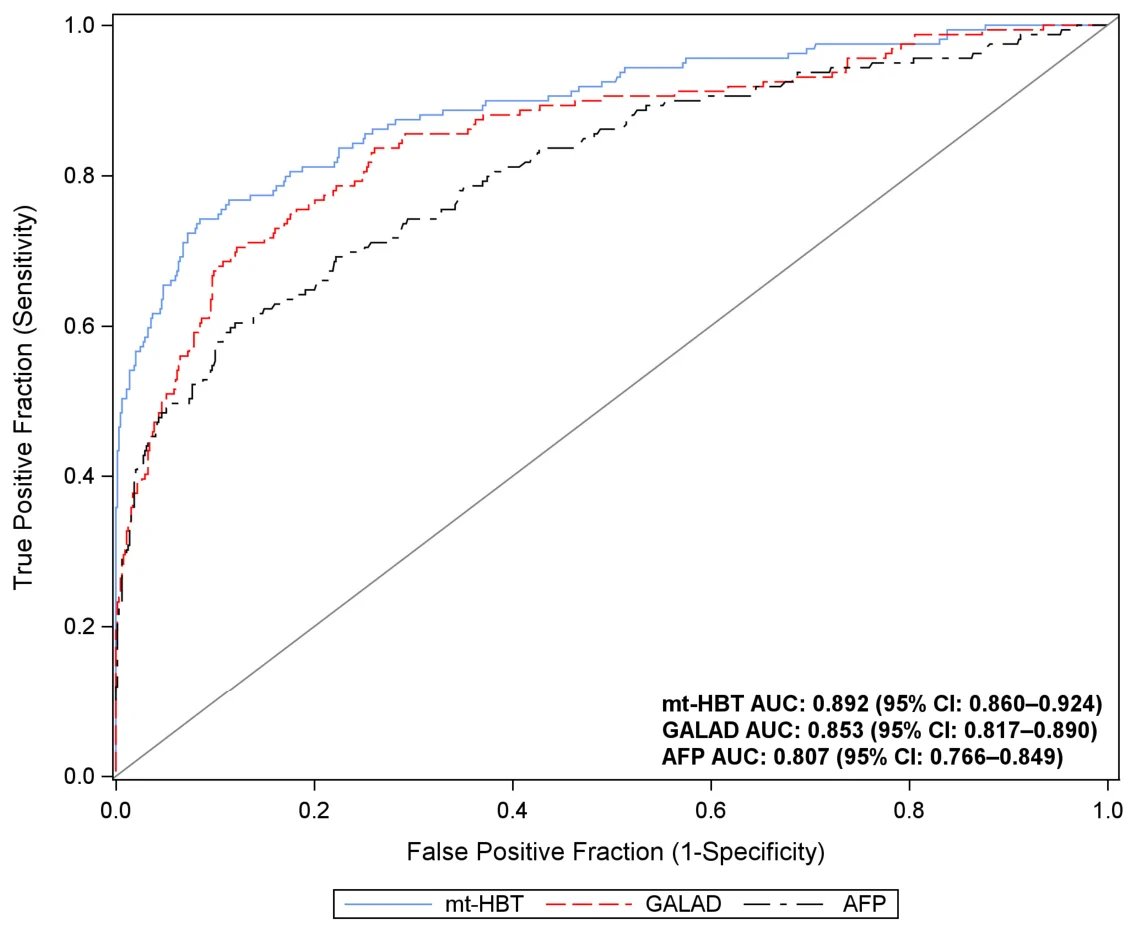
ROC Curves for mt-HBT, GALAD, and AFP in Detecting Early-Stage HCC versus Controls. AFP, alpha-fetoprotein; GALAD, Gender, Age, AFP-L3, AFP, and des–gamma–carboxy prothrombin; HCC, hepatocellular carcinoma; mt-HBT, multitarget hepatocellular carcinoma blood test; ROC, receiver operating characteristic.

Receiver operating characteristic analyses demonstrated that mt-HBT had the highest overall discriminatory performance for early-stage HCC detection, with an AUROC of 0.892 (95% CI, 0.860–0.924), compared with 0.853 (95% CI, 0.817–0.890) for GALAD and 0.807 (95% CI, 0.766–0.849) for AFP (Figure 1).

### 3.3. Sensitivity for Early-Stage HCC Across Subgroups

The sensitivity of mt-HBT was consistent across the examined subgroups, with no statistically significant differences, including populations in whom ultrasound performance was degraded (Table 2). Specifically, the sensitivity of mt-HBT for early-stage HCC was 81.6% (95% CI, 72.8–88.1) in patients with BMI < 30 compared with 68.9% (95% CI, 56.4–79.1) in those with BMI ≥ 30 (*p* = 0.06). In patients with Child Pugh A and B cirrhosis, mt-HBT sensitivity was 76.3% (95% CI, 67.7–83.2) and 75.0% (95% CI, 59.8–85.8), respectively (*p* = 0.87). Finally, mt-HBT had sensitivities of 80.0% (95% CI, 68.7–87.9) in hepatitis C, 72.2% (95% CI, 49.1–87.5) in HBV, 80.5% (95% CI, 66.0–89.8) in ALD, and 69.0 (95% CI 50.8–82.7) in MASLD (*p* = 0.35). Across all liver disease etiologies, the sensitivity of mt-HBT was significantly higher than that of AFP (*p* ≤ 0.01 for all) but did not significantly differ from GALAD (*p* > 0.05 for all). mt-HBT sensitivity was consistent in men (79.8%; 95% CI, 71.7–86.1) and women (67.5%; 95% CI, 52.0–79.9) as well as patients ≥ 65 years (74.3%; 95% CI, 63.0–83.1) and those <65 years (78.7%; 95% CI, 69.0–85.9) (*p* > 0.1 for both). mt-HBT had no significant difference in sensitivity compared with GALAD in women (*p* = 1.0), younger patients (*p* = 0.11), and men (*p* = 0.49), but lower sensitivity in older patients (*p* = 0.01).

### 3.4. Specificity Across Subgroups

The specificity of mt-HBT was consistent across examined subgroups, with no statistically significant differences, including populations in whom AFP is prone to false-positive results (Table 2). mt-HBT had specificities of 86.3% (95% CI 81.3–90.1) in hepatitis C, 94.4% (95% CI 87.5–97.6) in HBV, 84.4% (95% CI 77.1–89.7) in ALD, and 86.9 (95% CI 80.7–91.4) in MASLD (*p* = 0.20). The specificity of mt-HBT was 89.1% (95% CI 84.6–92.3) in patients ≥ 65 years compared to 86.5% (95% CI 82.8–89.5) in those <65 years (*p* = 0.34). The specificity of mt-HBT was 86.0% (95% CI 82.1–89.2) in patients with BMI < 30 compared to 89.5% (95% CI 85.4–92.6) in those with BMI ≥ 30 (*p* = 0.18). However, mt-HBT specificity appeared higher in women (92.6%, 95% CI 88.9–87.2) compared with men (83.9%, 95% CI 79.8–87.2%) (*p* < 0.001).

Across all liver disease etiologies, the specificity of mt-HBT was lower than that of AFP (*p* ≤ 0.01 for all). Specificity was higher than that of GALAD across all liver disease etiologies, particularly in those with MASLD or ALD. The specificity of mt-HBT was significantly higher than that of GALAD in men and older individuals (*p* < 0.001 for both) but did not significantly differ in women (*p* = 0.37) or patients < 65 years old (*p* = 0.58).

### 3.5. Sensitivity, Stratified by Maximum Tumor Diameter

The sensitivity of mt-HBT was proportional to tumor size, increasing from 60.0% (95% CI, 45.5–73) in patients with HCC < 2 cm to 77.4% (95% CI, 65.6–86.0) for lesions 2–3 cm, 82.1% (95% CI, 64.4–92.1) for lesions > 3 to 5 cm in maximum diameter, and 100% (95% CI, 86.2–100) for lesions > 5 cm.

### 3.6. Performance of Mt-HBT in Clinical Validation Sample Set

In the clinical validation sample set, the overall sensitivity and specificity of mt-HBT for early-stage HCC were 82.1% (95% CI, 72.1–89.0) and 86.9% (95% CI, 82.1–90.6), respectively (Table 3). Similar to the overall analysis, mt-HBT sensitivity did not significantly differ across most examined subgroups (Table 3). Notably, mt-HBT had sensitivities of 83.3% (95% CI, 68.1–92.1) in hepatitis C, 88.9% (95% CI, 56.5–98.0) in HBV, 87.0% (95% CI, 67.9–95.5) in ALD, and 66.7% (95% CI, 30.0–90.3) in MASLD. Across all liver disease etiologies, the sensitivity of mt-HBT was higher than that of AFP but did not differ significantly from GALAD. The sensitivity of mt-HBT continued to be proportional to tumor size, increasing from 65.0% (95% CI 43.3–81.9) in patients with HCC < 2 cm to 86.1% (95% CI, 71.3–93.9) for lesions 2–3 cm, 75.0% (95% CI, 40.9–92.9) for lesions > 3 to 5 cm in maximum diameter, and 100% (95% CI 78.5–100) for lesions > 5 cm. The specificity of mt-HBT was also consistent across subgroups without significant differences (Table 3). Indeed, the specificity of mt-HBT was higher than 80% in all examined subgroups and numerically higher than that of GALAD in patients with ALD, men, and older individuals.

## 4. Discussion

In summary, our results suggest that mt-HBT has consistently high sensitivity for early-stage HCC across patient subgroups. Notably, mt-HBT had preserved sensitivity in patients with obesity, non-viral liver disease, and Child Pugh class B cirrhosis—subgroups in whom ultrasound is prone to surveillance failure [17]. Further, it had acceptably high specificity, including significantly higher specificity than that of GALAD in men and older individuals. These data reinforce the diagnostic accuracy of mt-HBT across a wide range of patient subgroups observed in clinical practice.

In this analysis, we found that the sensitivity of mt-HBT was superior to that of AFP. Although we did not conduct direct comparisons with ultrasound in this study, its sensitivity for early-stage HCC compares favorably with that reported in a prior meta-analysis [15]. Further, the ALTUS Study recently demonstrated higher sensitivity of mt-HBT than that of ultrasound for early-stage HCC (66.7% vs. 22.2%) [28]. Although ALTUS provides prospective evaluation of ultrasound performance, the number of early-stage HCC cases was limited (*n* = 28), precluding subgroup analyses. Understanding how performance varies across these subgroups is important given the known variation in performance of other modalities. For example, ultrasound visualization and sensitivity are worse in patients with obesity and non-viral liver disease [17] AFP has lower sensitivity in non-viral liver disease [30]; and biomarker panels, such as GALAD, integrate age and sex into their algorithms and therefore have lower sensitivity in women and younger individuals [32]. Although sensitivity at the prespecified threshold did not significantly differ between mt-HBT and GALAD, ROC analyses demonstrated higher overall discriminatory performance with mt-HBT. The higher AUROC suggests mt-HBT may offer greater flexibility in threshold selection for different surveillance settings while maintaining favorable discrimination between patients with early-stage HCC and controls. These data are particularly important given changes in demographics and etiological patterns of HCC over time. There has been a global shift from viral hepatitis to MASLD and ALD as being the most common causes of HCC, particularly in the Western world [33,34]. Herein, we found that the sensitivity of mt-HBT was consistent across subgroups. The largest variation in sensitivity was observed in patients with and without obesity, with sensitivities of 68.9% and 81.6%, respectively. However, this difference did not reach statistical significance, and there is no strong scientific rationale for why the performance of methylated DNA panels would differ by BMI. Future studies will need to determine if there is a true difference in performance. Understanding these variations in sensitivity can inform precision surveillance strategies, in which surveillance strategies are not only determined by HCC risk but also by expected test performance. A recent decision analysis demonstrated the cost-effectiveness of precision surveillance compared with both risk-stratified and “one-size-fits-all” surveillance [35].

The sensitivity of mt-HBT is proportional to tumor burden, although sensitivity exceeded 60% in patients with T1 lesions between 1–2 cm. These data compare favorably with sensitivities from other studies of only ~28% for ultrasound at these small sizes [36]. Indeed, ALTUS demonstrated that mt-HBT sensitivity was 53.8% (95% CI, 29.1–76.8) compared with only 9.1% (95% CI, 1.6–37.7) for ultrasound plus AFP for detecting very early-stage HCC. Although early-stage HCC has long been considered the recommended outcome for surveillance studies, over one-third of patients with early-stage HCC continue to die from HCC-related causes [37]. Patients with early-stage HCC have significantly improved survival compared with patients with early-stage HCC, including those with HCC lesions 2–3 cm [38]. Although evaluation of very early-stage HCC was not informative with ultrasound-based surveillance, comparisons may be increasingly informative with emerging surveillance strategies. In addition to our study reinforcing the promise of methylated DNA panels to promote very early-stage HCC detection, studies of abbreviated MRI-based surveillance also demonstrate high sensitivity for very early-stage HCC [39,40,41]. Future real-world studies will need to determine if increased detection at the T1 tumor stage results in downstream improvements in survival, particularly in centers or regions where patients are monitored for the development of a T2 lesion for liver transplant priority status [42].

We also evaluated differences in specificity across patient subgroups. Although patients and providers place greater emphasis on benefits, including HCC detection, than screening-related harms, preserving adequate specificity is still essential for all cancer screening programs [43,44]. An expert stakeholder panel recently demonstrated a minimum threshold of 80% for specificity to minimize physical, financial, and psychological screening-related harms [45]. Although HCC surveillance results in minimal physical harms given the use of imaging instead of biopsy for diagnostic evaluation, there are potential psychological and financial harms that must be considered. These data are particularly important given recent data suggesting that some liquid biopsy techniques can detect preclinical disease, up to three years prior to clinical presentation [46]. Reassuringly, mt-HBT had preserved specificity exceeding the 80% threshold across all examined subgroups. Future studies will be required to determine longitudinal changes in mt-HBT to assess whether patients with false-positive results have normal results at the next interval or persistently positive results. If persistently abnormal, longitudinal changes in absolute values can inform provider practice patterns, including the need for and optimal intervals for repeat diagnostic testing. In addition, ongoing prospective real-world and post-marketing studies will provide opportunities to further evaluate mt-HBT performance across clinically relevant patient subgroups in independent patient cohorts, helping to validate the consistency of test performance observed in the current analysis.

Our study’s limitations include its case-control design, with inherent limitations including the inability to calculate positive and negative predictive values, spectrum bias, and overestimation of test performance. Performance may also be overestimated given the inclusion of data from the algorithm development and clinical validation sample sets. However, results were consistent in the subgroup comprising the clinical validation sample set alone. Further, these concerns are mitigated by the alignment of test performance in this study with that reported in ALTUS, particularly when stratified by tumor size. Small sample sizes produced wide confidence intervals in some subgroups, which may preclude definitive conclusions, particularly when comparing the performance of mt-HBT to that of AFP and GALAD. Finally, although we compared mt-HBT performance with other biomarkers, we did not have data to compare mt-HBT with abdominal ultrasound. However, we feel these limitations are outweighed by the study’s strengths, including its large overall sample size, concurrent testing of both AFP and GALAD allowing head-to-head comparisons, and a contemporary patient population with the majority having non-viral liver disease.

## 5. Conclusions

mt-HBT is a promising blood-based biomarker for early-stage HCC detection, with consistent performance across several patient subgroups, supporting its ongoing prospective validation for HCC surveillance.

## Figures and Tables

**Table 1:** Patient Characteristics.

Patient Characteristic	Total Patients (*N* = 808)	Early-Stage HCCs (*N* = 159)	Controls (*N* = 649)
Age			
Mean (SD)	62.9 (7.95)	64.1 (8.09)	62.6 (7.90)
Median (min, max)	63 (29, 85)	64 (35, 84)	62 (29, 85)
Gender			
Female	311 (38.5%)	40 (25.2%)	271 (41.8%)
Male	497 (61.5%)	119 (74.8%)	378 (58.2%)
Etiology			
Hepatitis C	298 (36.9%)	65 (40.9%)	233 (35.9%)
MASLD	182 (22.5%)	29 (18.2%)	153 (23.6%)
Alcohol-associated	169 (20.9%)	41 (25.8%)	128 (19.7%)
Hepatitis B	107 (13.2%)	18 (11.3%)	89 (13.7%)
Other	52 (6.4%)	6 (3.8%)	46 (7.1%)
BMI			
≥30	338 (41.8%)	61 (38.4%)	277 (42.7%)
<30	470 (58.2%)	98 (61.6%)	372 (57.3%)
Child Pugh Class			
A	599 (74.1%)	114 (71.7%)	485 (74.7%)
B	177 (21.9%)	40 (25.2%)	137 (21.1%)
Missing	32 (4.0%)	5 (3.1%)	27 (4.2%)
Cirrhosis Status			
Yes	753 (93.2%)	153 (96.2%)	600 (92.4%)
No	55 (6.8%)	6 (3.8%)	49 (7.6%)

**Table 2:** Performance of mt-HBT, Overall and in Subgroups.

Performance Characteristic	mt-HBT	AFP (20 ng/mL)	GALAD (−1.36)
**Overall**			
Sensitivity, % (95% CI) (*n* = 159)	76.7 (69.6–82.6%)	35.2 (28.2–42.9%)	78.6 (71.6–84.3%)
Specificity, % (95% CI) (*n* = 649)	87.5 (84.8–89.8%)	98.5 (97.2–99.2%)	76.9 (73.5–80.0%)
AUC (95% CI)	89.2 (86.0–92.4%)	80.7 (76.6–84.9%)	85.3 (81.7–0.89%)
**Hepatitis C Virus**			
Sensitivity, % (95% CI) (*n* = 65)	80.0 (68.7–87.9%)	50.8 (38.9–62.5%)	78.5 (67.0–86.7%)
Specificity, % (95% CI) (*n* = 233)	86.3 (81.3–90.1%)	97.4 (94.5–98.8%)	79.0 (73.3–83.7%)
**Hepatitis B Virus**			
Sensitivity, % (95% CI) (*n* = 18)	72.2 (49.1–87.5%)	27.8 (12.5–50.9%)	66.7 (43.7–83.7%)
Specificity, % (95% CI) (*n* = 89)	94.4 (87.5–97.6%)	100.0 (95.9–100.0%)	91.0 (83.3–95.4%)
**Alcohol-associated liver disease**			
Sensitivity, % (95% CI) (*n* = 41)	80.5 (66.0–89.8%)	26.8 (15.7–41.9%)	80.5 (66.0–89.8%)
Specificity, % (95% CI) (*n* = 128)	84.4 (77.1–89.7%)	98.4 (94.5–99.6%)	64.1 (55.5–71.9%)
**Metabolic dysfunction associated with steatotic liver disease**			
Sensitivity, % (95% CI) (*n* = 29)	69.0 (50.8–82.7%)	20.7 (9.8–38.4%)	82.8 (65.5–92.4%)
Specificity, % (95% CI) (*n* = 153)	86.9 (80.7–91.4%)	98.7 (95.4–99.6%)	74.5 (67.1–80.8%)
**Child Pugh A Cirrhosis**			
Sensitivity, % (95% CI) (*n* = 114)	76.3 (67.7–83.2%)	32.5 (24.6–41.5%)	76.3 (67.7–83.2%)
Specificity, % (95% CI) (*n* = 485)	88.9 (85.8–91.4%)	98.6 (97.1–99.3%)	81.9 (78.2–85.0%)
**Child Pugh B Cirrhosis**			
Sensitivity, % (95% CI) (*n* = 40)	75.0 (59.8–85.8%)	42.5 (28.5–57.8%)	85.0 (70.9–92.9%)
Specificity, % (95% CI) (*n* = 137)	81.8 (74.4–87.3%)	97.8 (93.8–99.3%)	57.7 (49.3–65.6%)
**Patients without Obesity**			
Sensitivity, % (95% CI) (*n* = 98)	81.6 (72.8–88.1%)	37.8 (28.8–47.6%)	79.6 (70.6–86.4%)
Specificity, % (95% CI) (*n* = 372)	86.0 (82.1–89.2%)	97.6 (95.5–98.7%)	76.6 (72.1–80.6%)
**Patients with Obesity**			
Sensitivity, % (95% CI) (*n* = 61)	68.9 (56.4–79.1%)	31.1 (20.9–43.6%)	77.0 (65.1–85.8%)
Specificity, % (95% CI) (*n* = 277)	89.5 (85.4–92.6%)	99.6 (98.0–99.9%)	77.3 (72.0–81.8%)
**Male Sex**			
Sensitivity, % (95% CI) (*n* = 119)	79.8 (71.7–86.1%)	32.8 (25.0–41.6%)	82.4 (74.5–88.2%)
Specificity, % (95% CI) (*n* = 378)	83.9 (79.8–87.2%)	98.7 (96.9–99.4%)	66.9 (62.0–71.5%)
**Female Sex**			
Sensitivity, % (95% CI) (*n* = 40)	67.5 (52.0–79.9%)	42.5 (28.5–57.8%)	67.5 (52.0–79.9%)
Specificity, % (95% CI) (*n* = 271)	92.6 (88.9–95.2%)	98.2 (95.8–99.2%)	90.8 (86.7–93.7%)
**Age ≥ 65 Years**			
Sensitivity, % (95% CI) (*n* = 70)	74.3 (63.0–83.1%)	30.0 (20.5–41.5%)	87.1 (77.3–93.1%)
Specificity, % (95% CI) (*n* = 256)	89.1 (84.6–92.3%)	98.4 (96.1–99.4%)	64.1 (58.0–69.7%)
**Age < 65 Years**			
Sensitivity, % (95% CI) (*n* = 89)	78.7 (69.0–85.9%)	39.3 (29.8–49.7%)	71.9 (61.8–80.2%)
Specificity, % (95% CI) (*n* = 393)	86.5 (82.8–89.5%)	98.5 (96.7–99.3%)	85.2 (81.4–88.4%)
**Tumor Size < 2 cm**			
Sensitivity, % (95% CI) (*n* = 45)	60.0 (45.5–73.0%)	28.9 (17.7–43.4%)	64.4 (49.8–76.8%)
**Tumor Size 2–3 cm**			
Sensitivity, % (95% CI) (*n* = 62)	77.4 (65.6–86.0%)	27.4 (17.9–39.6%)	82.3 (71.0–89.8%)
**Tumor Size 3–5 cm**			
Sensitivity, % (95% CI) (*n* = 28)	82.1 (64.4–92.1%)	46.4 (29.5–64.2%)	82.1 (64.4–92.1%)
**Tumor Size ≥ 5 cm**			
Sensitivity, % (95% CI) (*n* = 24)	100.0 (86.2–100.0%)	54.2 (35.1–72.1%)	91.7 (74.2–97.7%)

**Table 3:** Performance of mt-HBT, Overall and in Subgroups in Clinical Validation Sample Set.

Performance Characteristic	mt-HBT	AFP (20 ng/mL)	GALAD (−1.36)
**Overall**			
Sensitivity, % (95% CI) (*n* = 78)	82.1 (72.1–89.0%)	39.7 (29.6–50.8%)	79.5 (69.2–87.0%)
Specificity, % (95% CI) (*n* = 245)	86.9 (82.1–90.6%)	100.0 (98.5–100.0%)	83.3 (78.1–87.4%)
**Hepatitis C Virus**			
Sensitivity, % (95% CI) (*n* = 36)	83.3 (68.1–92.1%)	52.8 (37.0–68.0%)	83.3 (68.1–92.1%)
Specificity, % (95% CI) (*n* = 93)	88.2 (80.1–93.3%)	100.0 (96.0–100.0%)	88.2 (80.1–93.3%)
**Hepatitis B Virus**			
Sensitivity, % (95% CI) (*n* = 9)	88.9 (56.5–98.0%)	44.4 (18.9–73.3%)	55.6 (26.7–81.1%)
Specificity, % (95% CI) (*n* = 33)	93.9 (80.4–98.3%)	100.0 (89.6–100.0%)	87.9 (72.7–95.2%)
**Alcohol-associated liver disease**			
Sensitivity, % (95% CI) (*n* = 23)	87.0 (67.9–95.5%)	30.4 (15.6–50.9%)	82.6 (62.9–93.0%)
Specificity, % (95% CI) (*n* = 51)	80.4 (67.5–89.0%)	100.0 (93.0–100.0%)	68.6 (55.0–79.7%)
**Metabolic dysfunction associated with steatotic liver disease**			
Sensitivity, % (95% CI) (*n* = 6)	66.7 (30.0–90.3%)	0.0 (0.0–39.0%)	83.3 (43.6–97.0%)
Specificity, % (95% CI) (*n* = 49)	85.7 (73.3–92.9%)	100.0 (92.7–100.0%)	83.7 (71.0–91.5%)
**Child Pugh A Cirrhosis**			
Sensitivity, % (95% CI) (*n* = 52)	80.8 (68.1–89.2%)	34.6 (23.2–48.2%)	78.8 (66.0–87.8%)
Specificity, % (95% CI) (*n* = 168)	87.5 (81.6–91.7%)	100.0 (97.8–100.0%)	86.9 (81.0–91.2%)
**Child Pugh B Cirrhosis**			
Sensitivity, % (95% CI) (*n* = 21)	81.0 (60.0–92.3%)	52.4 (32.4–71.7%)	81.0 (60.0–92.3%)
Specificity, % (95% CI) (*n* = 53)	83.0 (70.8–90.8%)	100.0 (93.2–100.0%)	71.7 (58.4–82.0%)
**Patients without Obesity**			
Sensitivity, % (95% CI) (*n* = 48)	85.4 (72.8–92.8%)	39.6 (27.0–53.7%)	79.2 (65.7–88.3%)
Specificity, % (95% CI) (*n* = 141)	86.5 (79.9–91.2%)	100.0 (97.3–100.0%)	80.9 (73.6–86.5%)
**Patients with Obesity**			
Sensitivity, % (95% CI) (*n* = 30)	76.7 (59.1–88.2%)	40.0 (24.6–57.7%)	80.0 (62.7–90.5%)
Specificity, % (95% CI) (*n* = 104)	87.5 (79.8–92.5%)	100.0 (96.4–100.0%)	86.5 (78.7–91.8%)
**Male Sex**			
Sensitivity, % (95% CI) (*n* = 65)	83.1 (72.2–90.3%)	38.5 (27.6–50.6%)	81.5 (70.4–89.1%)
Specificity, % (95% CI) (*n* = 144)	84.0 (77.2–89.1%)	100.0 (97.4–100.0%)	76.4 (68.8–82.6%)
**Female Sex**			
Sensitivity, % (95% CI) (*n* = 13)	76.9 (49.7–91.8%)	46.2 (23.2–70.9%)	69.2 (42.4–87.3%)
Specificity, % (95% CI) (*n* = 101)	91.1 (83.9–95.2%)	100.0 (96.3–100.0%)	93.1 (86.4–96.6%)
**Age ≥ 65 Years**			
Sensitivity, % (95% CI) (*n* = 30)	86.7 (70.3–94.7%)	33.3 (19.2–51.2%)	93.3 (78.7–98.2%)
Specificity, % (95% CI) (*n* = 63)	88.9 (78.8–94.5%)	100.0 (94.3–100.0%)	71.4 (59.3–81.1%)
**Age < 65 Years**			
Sensitivity, % (95% CI) (*n* = 48)	79.2 (65.7–88.3%)	43.8 (30.7–57.7%)	70.8 (56.8–81.8%)
Specificity, % (95% CI) (*n* = 182)	86.3 (80.5–90.5%)	100.0 (97.9–100.0%)	87.4 (81.8–91.4%)
**Tumor Size < 2 cm**			
Sensitivity, % (95% CI) (*n* = 20)	65.0 (43.3–81.9%)	40.0 (21.9–61.3%)	65.0 (43.3–81.9%)
**Tumor Size 2–3 cm**			
Sensitivity, % (95% CI) (*n* = 36)	86.1 (71.3–93.9%)	33.3 (20.2–49.7%)	86.1 (71.3–93.9%)
**Tumor Size 3–5 cm**			
Sensitivity, % (95% CI) (*n* = 8)	75.0 (40.9–92.9%)	50.0 (21.5–78.5%)	75.0 (40.9–92.9%)
**Tumor Size ≥ 5 cm**			
Sensitivity, % (95% CI) (*n* = 14)	100.0 (78.5–100.0%)	50.0 (26.8–73.2%)	85.7 (60.1–96.0%)

## Data Availability

All data generated or analyzed during this study are included in this article. Further inquiries can be directed to clinicaltrials@exactsciences.com.
